# Systematic searching: practical ideas for improving results

**DOI:** 10.29173/jchla29705

**Published:** 2023-08-01

**Authors:** Genevieve Gore

**Affiliations:** Associate Librarian, Schulich Library of Physical Sciences, Life Sciences, and Engineering McGill University, Montreal Quebec, Canada

Levay P, Craven J, editors. **Systematic searching: practical ideas for improving results**. London: Facet Publishing; 2019. Softcover: 320 p. ISBN: 978-1-78330-373-1. Price: USD$94.99. Available from: https://www.facetpublishing.co.uk/page/detail/systematic-searching/?k=9781783303731

*Systematic searching: practical ideas for improving results* is an accessible and pragmatic guide to advanced search techniques and issues. Published in the UK by Facet Publishing, which is owned by the Chartered Institute of Library and Information Professionals, *Systematic searching* covers methods and techniques, innovative technologies, and the people involved in the process of developing systematic searches. Edited by Paul Levay and Jenny Craven from the National Institute for Health and Care Excellence, this book will interest searchers not only in health and biological sciences, but also those advancing research in the social sciences and beyond.

Each chapter is authored by at least one noteworthy researcher or practitioner. Their names are worth mentioning as readers with a familiarity of the literature will recognize them from studies published on searching methodology and search strategy development.

In chapter two, Andrew Booth describes some of the contexts in which innovative approaches would be deemed acceptable as well as appropriate, touching upon controversy related to sampling and search efficiency. Booth addresses the need to understand the implications of different review types on searching (for example, the role of iterative searching in realist reviews, or the role of theory or framework searching). Given the predominant role of systematic review methodologies in the literature, it is easy to forget that searching requires an understanding of context. Those not familiar with the large array of question structure frameworks will also benefit from a reading of this chapter, although not all question structures are covered, for example PCC (Population, Context, and Concept) for scoping reviews.

In chapter three, “searching for broad-based topics,” Claire Stansfield discusses issues faced in reviews informing public policy decisions and provides practical approaches to handling broad-based topics. This is another area that may be overlooked in education and training. Alison Bethel and Morwenna Rogers then discuss database selection and other search techniques in chapter four. The authors provide an overview of the literature on the number of databases needed to search and recommend search summary tables which are used at the completion of a review to allow the searcher to see which databases retrieved which included studies. The chapter quickly covers other search techniques such as citation searching and handsearching. It touches on the selection of smaller databases, described as often containing valuable sources of grey literature. Shannon Kugley and Richard Epstein further the discussion on grey literature in chapter five. Although focused on health sciences and policy development, the authors discuss the lack of guidance on how to search, incorporate, appraise, and document grey literature. Su Golder then delves into the potential uses of social media in chapter six, breaking down its uses in original ways.

Chapter seven explores text mining. Written by Julie Glanville, it provides a trove of examples of the application of text mining to searching. Glanville suggests techniques that are clearly documented through easy-to-follow examples and screenshots (albeit sometimes a bit blurry). A case is clearly made for the technique’s utility in understanding a topic, identifying terms (including phrases), developing search filters, identifying relevant papers, identifying concepts, and screening. Table 7.1 will be of interest to anyone wishing to see a list of tools by task. The clear illustrations of the applied use of a range of tools will benefit any searcher with or without text mining experience.

Andy Mitchell and Chris Mavergames tackle the tricky topic of linked data in chapter eight, providing the concrete example of the Cochrane Linked Data Project, and encouraging further readings. James Thomas, Anna Noel-Storr, and Steve McDonald follow with a discussion of automation in the context of evidence surveillance and living systematic reviews. They provide case studies of the Cochrane Evidence Pipeline and the Human Behaviour-Change Project. The chapter contains many references to automated and semi-automated approaches.

Chapters ten through thirteen, authored by Michelle Maden, Gil Young, Siw Waffenschmidt, Elke Hausner, Margaret Sampson, and Alison Brettle, address training, collaboration, communication, and the roles of expert searchers. Maden and Young nicely summarize the competencies required for systematic searchers and emphasize the need for training that is specific to review types, as well as the role of attitudes (such as confidence) and other contextual knowledge and skills in searching. They also present a case for an international review of expert searcher education by health library organizations and the potential role of accreditation. Waffenschmidt and Hausner then shift the discussion to opportunities for information specialists to collaborate on review teams, mentioning the importance of the review protocol and how this can be used as a tool to clarify roles and responsibilities. Sampson emphasizes the importance of communication as a core professional competency for collaborators and within scholarly communities, and Brettle reviews the roles of expert searchers.

Although this book was published in 2019, novice and advanced searchers will find guidance that may not yet arguably be considered mainstream, which can be a source of reassurance and confidence building when approaching alternative techniques for searching. The contents provide a solid foundation or reminder of some of the less-straightforward searching issues that are likely to be encountered, particularly when dealing with questions that include broader or more complex concepts. These include the role of comprehensiveness versus representativeness in search results for broad-based questions and the role of sampling in qualitative evidence synthesis. Both are topics that may not be sufficiently addressed in training on systematic searching.

Most of the chapters use case studies to illustrate applications or points. I would have loved to see some additional guidance in areas in which the authors explicitly mention formal guidance to be lacking. I appreciated the chapter on text mining with its practical instructions on when and how to use the tools being suggested. The chapter on linked data, although interesting, was difficult for me to translate into practical uses in systematic searching. That said, it is an important topic to understand. Similarly, many librarians and information specialists could benefit from a better understanding of the technology behind automation tools. A few chapters incorporate information on artificial intelligence in searching (chapters seven, eight, and nine in particular). Here I would have liked to see more content on natural language processing and machine learning. These are topics that searchers would do well to understand at a basic level, given how many of the resources currently used incorporate or build upon such approaches. I would predict that any future edition of this title, which I hope will be published, would incorporate more information on the potential uses of PubMed’s “best match” and “similar articles” functions in systematic searching, new tools for mining full text, and large language models such as ChatGPT. Even though a lot of the information is still relevant today, the fast-paced developments in these areas underscore the importance of keeping up to date with innovative approaches in searching, a point which is indeed emphasized in this well-resourced and well-referenced book. Having purchased this title with professional development funds a few years ago, I was grateful to have the opportunity to fully ascertain that its acquisition was worthwhile.

